# Association of child weight and adverse outcomes following antibiotic prescriptions in children: a national data study in Wales, UK

**DOI:** 10.1136/bmjpo-2024-002831

**Published:** 2024-11-28

**Authors:** Ayodele Vincent Opatola, Mike J Seaborne, Jonathan Kennedy, Dyfrig Hughes, Hamish Laing, Rhiannon K Owen, David Tuthill, Robert Bracchi, Sinead Brophy

**Affiliations:** 1 National Centre for Population Health and Wellbeing Research, Swansea University, Swansea, Wales, UK; 2 Centre for Health Economics and Medicines Evaluation, Bangor University, Bangor, Wales, UK; 3 School of Management, Swansea University, Swansea, Wales, UK; 4 medical statistics, Swansea University, Swansea, Wales, UK; 5 Paediatrics, Children's hospital for Wales, Cardiff, Cardiff, UK; 6 NHS All Wales Therapeutics and Toxicology Centre, Llandough, UK

**Keywords:** Statistics, Infant, Health Policy

## Abstract

**Objective:**

To examine if the weight of a child determines adverse events following oral antibiotics prescription.

**Design:**

Population respective cohort using linked general practice (GP), hospital data and linkage with the Welsh Demographic Service for demographic information. Data linkage was performed using Wales health data, extracted from the SAIL (Secure Anonymised Information Linkage) databank.

**Inclusion:**

Children (0–12 years) prescribed oral antibiotics by their GP in Wales.

**Exposure:**

Antibiotic prescription (penicillins, cephalosporins, macrolides, dihydropyrimidines, nitroimidazoles, nitrofurans, lincosamides).

**Outcome:**

Adverse event as defined by; patients’ death within 5 days, records of emergency admission within 5 days and GP records of adverse drug reactions or prescription of another antibiotic within 14 days.

**Analysis:**

Logistic regression of adverse events versus no adverse events at follow-up time.

**Results:**

There were 139 571 prescriptions of the selected antibiotics and 71 541 children (51.39% male) included with follow-up data of which there were 25 445 (18.23% of all prescriptions) children experienced adverse outcomes. There was higher odds of adverse events for lower weight children and those who were younger, female, of Asian origin or deprived.

**Conclusion:**

The findings support the hypothesis that smaller children for their age (eg, low weight, female, Asian) are more likely to experience adverse events following antibiotics prescription. This work suggests child weight, in addition to age, should be used when prescribing antibiotics to children in primary care.

**WHAT IS ALREADY KNOWN ON THIS TOPIC**

**WHAT THIS STUDY ADDS**

- This study reveals that low-weight children, females, and minority ethnic groups face higher risks of adverse events following oral antibiotic prescriptions.

**HOW THIS STUDY MIGHT AFFECT RESEARCH, PRACTICE OR POLICY**

- The findings suggest revising paediatric antibiotic prescribing guidelines to prioritize weight measurements, aiming to enhance dosing accuracy and reduce adverse outcomes in children.

## Introduction

### Background

 The escalating concern over antimicrobial resistance has prompted increased scrutiny of antibiotic prescription practices worldwide.^1^ Striking a delicate equilibrium between safety and efficacy holds utmost significance when administering antibiotics to children, as any deviation from this balance can lead to unwanted consequences.^2^ Selecting antibiotics based on a recognised formulary, tailoring dosages to individual patient characteristics and considering adverse drug reactions specific to each patient are crucial considerations in paediatric antibiotic therapy. More than one-third of British children annually undergo antibiotic therapy, with oral penicillins constituting a substantial majority. They are frequently prescribed to address common respiratory tract infections.^3^,^5^ While most antibiotics have a low risk-to-benefit ratio for infectious illnesses,^6^ appropriate dosing is important.

The practice of prescribing oral penicillins as fractions of adult doses in children’s age groups was established in the 1960s and maintained until 2011 when concerns were raised about suboptimal dosing of amoxicillin for overweight children.^7^ Prescribing recommendations underwent revision in 2014 because of concerns about potential under-dosing.^8^ In 2014, the dosage was increased twofold in all age groups.^9^

Paediatric drug dosing often demands precision with consideration of both age/development and weight. The British National Formulary for Children (BNFC)^10^ details an age-banded system for most commonly prescribed oral antibiotics in primary care. This simplifies prescribing by eliminating the need for real-time weight measurement. However, this could lead to suboptimal dosing due to the non-linear relationship between age and weight in children.^11^ Age and weight necessitate consistent documentation and special attention in paediatric antibiotic prescriptions due to distinct growth trajectories compared with adults.^12^ In continental Europe, prescriptions are typically weight-based, offering a potentially more tailored approach. Given that boys generally have higher average weights than girls,^13^ and children’s weights exhibit significant variability^14^; individualised dosing that considers both age and weight is crucial to the safe prescribing of antibiotics. It would likely result in meeting more of the antibiotics’ therapeutic indices.^15^ This necessitates a focused evaluation of dosing strategies to enhance accuracy in paediatric pharmacotherapy.

## Objective

This study examines the association of adverse outcomes associated with oral antibiotic prescribing practices in paediatric primary care in Wales, with a specific emphasis on child weight. It examines major factors such as the age bands of children (based on the BNFC guidance), weight categories (grouped by centiles for sex and age), ethnicity, deprivation quintile and sex. Our study employs a sophisticated statistical approach known as a multilevel multivariate logistic regression model.^16^ This model is tailored to handle within-patient correlation and heterogeneity, which is crucial given that multiple records for individual patients are present within our study period. Specifically, we aim to investigate the likelihood of adverse events following oral antibiotic prescriptions in general practice (GP).

## Method

### Sample selection

**Figure 1:**
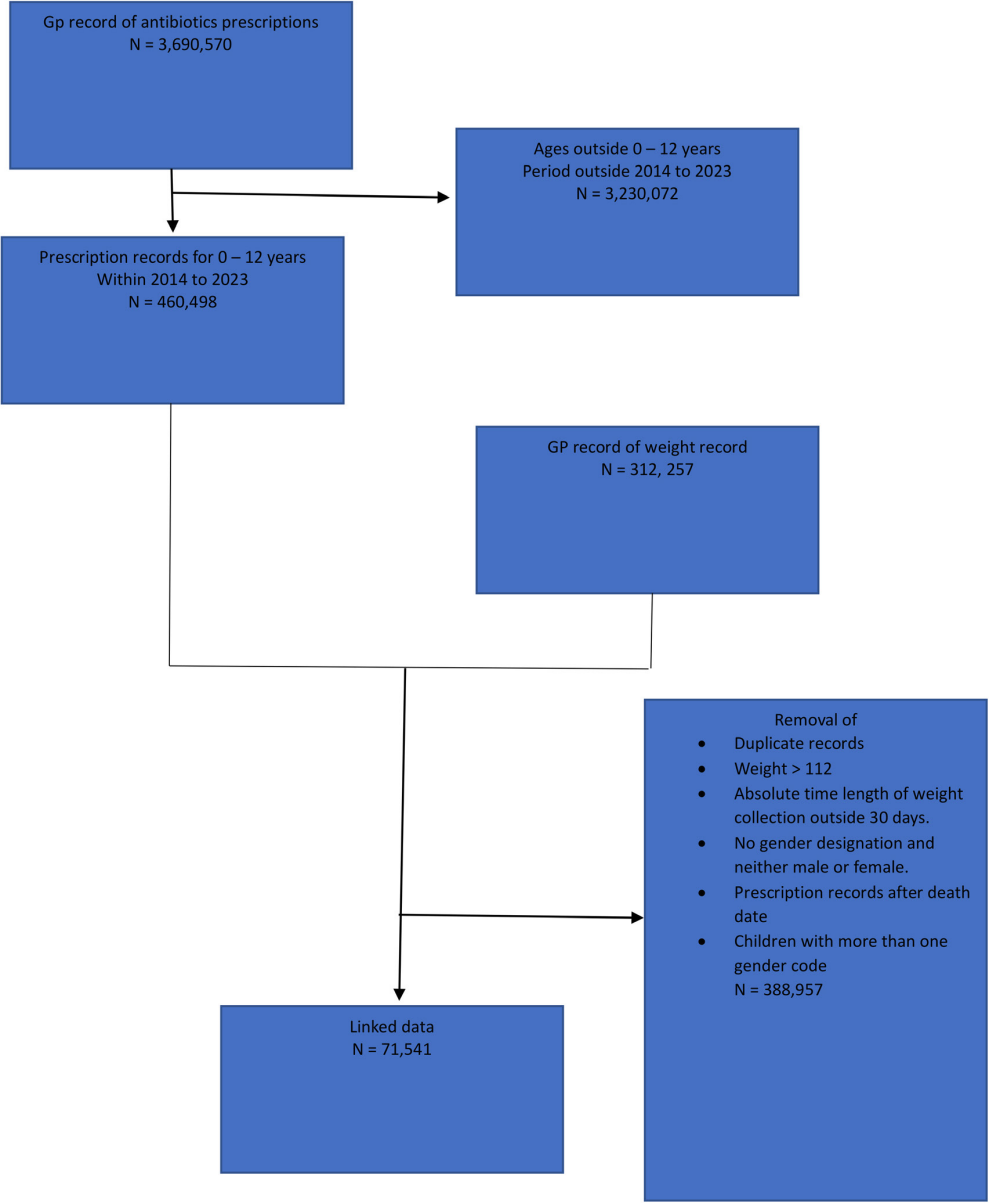
Flow chart showing inclusion and exclusions from Welsh Longitudinal General Practice data set and National Community Child Health Database. GP, general practice.

In this retrospective cohort study, we used routinely-collected GP prescription data for antibiotics prescribed for children in Wales between the period of January 2014 and October 2023. Prescriptions were identified using Read Codes (V.2). The list of codes used are available in online supplemental appendix 1.^17^ The inclusion criteria for the study included children between the ages of 0 and 12 years within the study period who had been issued with primary care prescription for oral antibiotics. Child weight data from Welsh Longitudinal General Practice (WLGP) data set were linked using the reference. Records with erroneous weights were excluded. Weights were considered erroneous if they were greater than 112 kg or were recorded more than 30 days before or after the oral antibiotics prescription date. The data linkage was carried out using the encrypted Anonymised Linking Field encrypted key in the Secure Anonymised Information Linkage (SAIL) databank.^18^ The antibiotics studied include common oral antibiotics classes used in children such as beta-lactams (penicillins and cephalosporins), macrolides, dihydropyrimidines (trimethoprim), nitroimidazole (metronidazole), nitrofuran (nitrofurantoin) and lincosamides. A flow diagram of the cohort selection can be found in figure 1.

### Risk factors and data linkage

Patient demographic information such as age and gender were linked from the WLGP data set; deprivation quintile data was linked from the Welsh Demographic Service Dataset^19^; patient ethnicity data was linked from the Patient Episode Dataset for Wales^20^; and, patient birth weight data was linked from the National Community Child Health Database.^21^ A brief description of the risk factors and their sources can be found in online supplemental appendix 2. This study acknowledges the multifaceted nature of paediatric antibiotic therapy and specifically focuses on key determinants, including: (1) Deprivation quintile: Given that socioeconomic inequalities exist and can be a major problem in appropriate healthcare delivery on a national scale.^22^ For this, we used a quintile categorisation of populations into five groups based on their Welsh Index of Multiple Deprivation scores. These quintiles are used to represent different levels of deprivation, with the first quintile representing the least deprived areas and the fifth quintile representing the most deprived areas. (2) Ethnicity: Knowledge and use of antibiotics have been shown to differ in different ethnic groups.^23^ (3) Sex: There are physiological and anatomical differences between males and females, this could influence the pharmacology of the prescribed antibiotics in the respective sexes.^24 25^ (4) Weight categories: The weight categories used were: low weight category (LWC grouped by sex and age group; with weights equal or less than the 25th percentile), normal weight category (NWC grouped by sex and age group; with weights above the 25th percentile and less than the 75th percentile) and, high weight category (HWC grouped by sex and age group; with weights equal or greater than the 75th percentile). And, (5) Age bands: The age band categories studies were 0–28 days (neonates), 1–11 months, 1–4 years and, 5–12 years. These represent the age bands in which children are often grouped during GP antibiotics prescription, based on the BNFC. No imputation techniques were applied to the variables in this study to handle missing values. This decision was made to maintain the representativeness of the sample and avoid introducing assumptions.

Not Available (NA) values for deprivation quintiles and ethnicity were categorised under a separate category labelled as missing.

### Adverse events identification

Four binary foundation phase indicator variables were derived from the linked data set; however, no formal assessment of causality was carried out. These include: (1) Patient death identified within 5 days of the initial antibiotic prescription; (2) repeated antibiotic prescribing within 14 days of an initial antibiotic prescription; (3) non-elective hospital/emergency admission within 5 days of antibiotics prescription; and, (4) GP record of toxicity, poisoning, overdose, allergy or hypersensitivity reactions within 14 days of antibiotics prescription (Read Codes V.2 used to identify these events in the WLGP data set can be found in online supplemental appendix 3). The data source used to generate these adverse events can be found in online supplemental appendix 4.

### Statistical analysis

A multilevel logistic regression model was used to measure the associated weight of each risk factor to the general adverse events outcome (as well as certain specific adverse event outcome based on availability of the sufficient oral antibiotics prescription data). A sensitivity analysis was performed on the data, which included records that were more than 30 days before or after the antibiotic prescription date and weight values above 112 kg. This analysis aimed to assess the impact of using potentially erroneous weight values for the children. Additional details can be found in online supplemental appendix 5. Data preparation was carried out on a DB2 SQL platform and the statistical analysis was performed on R V.4.0.3. using the following libraries: RODBC,^26^ tidyverse,^27^ lubridate^28^ and caret.^29^

### Logistic regression

We conducted a multilevel logistic regression for all the outcomes using the factors of interest as the covariates. The regression model was applied to generate OR plots, using the normal weight category as the reference in the weight category column, the highest quintile (deprivation quintile 5) as the reference for the deprivation quintiles column, white ethnicity compared with all other ethnicities in the ethnic group column and the 1–4 years age band compared with all other age bands in the age band column. These categories were selected as references based on the fact that they were the most common groups in their respective categories. The risk factors of adverse events following oral antibiotics prescription were presented with an adjusted OR (aOR) (adjusted for age band, weight category, sex, deprivation quintile and ethnicity) and 95% CI.

### Ethical considerations

Due to the anonymity of the data which is specifically collated by SAIL for research purposes, no additional ethical approval of this research was required.^30^

### Patient and public involvement

Patient and public involvement (PPI) was not directly incorporated into the design or conduct of this study. The data used for the design and implementation of this analysis was obtained from the SAIL databank, subject to approval from its IGRP which includes members of the public.

We recognise the most effective way to make the findings of this research relevant, accessible and impactful is to involve individuals and organisations which directly interface with these issues. For the dissemination of our findings, we plan to collaborate with the National Centre for Population Health and Well-being Research to involve their PPI group in interpreting the findings, identifying key messages and advising how best to communicate with relevant charities and organisations, such as the Children’s Commissioner for Wales. Additionally, we will seek the expertise of Dr David Tuthill, a consultant paediatrician at the Children’s Hospital for Wales in Cardiff, to facilitate outreach and promote awareness of our results among healthcare professionals.

## Results

### Sample characteristics

The study comprised 71 541 children meeting the inclusion criteria of a GP prescription for oral antibiotics (there were 139 571 prescriptions associated with 25 445 (18.2% of all) general adverse drug outcomes.), coupled with a weight record from WLGP within 30 days of prescription. Of these, 36 762 were boys, among whom 21.3% experienced at least one adverse event and 34 779 were girls, with 23.1% experiencing at least one adverse event. Among the participants, 22 140 fell into the LWC, with 21.0% experiencing at least one adverse event, while 37 240 were categorised as NWC, among whom 21.1% experienced at least one adverse event. Additionally, 22 778 children were classified as HWC, with 20.0% experiencing at least one adverse event. The overall summary of the study population can be found in table 1.

**Table 1:** Demographic data for the study cohort

	Total number	Total all outcomes N (%)	Total repeat antibiotics N (%)	Total hospital/emergency admission N (%)
Sex				
Female	34 779	8037 (23.11)	7165 (20.60)	1455 (4.18)
Male	36 762	7846 (21.34)	6791 (18.47)	1737 (4.72)
Age bands				
0–28 days	442	55 (12.44)	32 (7.24)	24 (5.43)
1–11 months	10 333	2051 (19.85)	1557 (15.07)	704 (6.81)
1–4 years	27 295	6670 (24.44)	5809 (21.28)	1413 (5.18)
5–12 years	41 041	7862 (19.40)	7238 (17.64)	1146 (2.79)
Deprivation quintiles				
1	18 133	3926 (21.65)	3412 (18.82)	840 (4.63)
2	14 158	3043 (21.49)	2670 (18.86)	629 (4.44)
3	12 038	2636 (21.90)	2139 (19.51)	482 (4.00)
4	10 636	2392 (22.49)	2145 (20.17)	389 (3.66)
5	10 829	2371 (21.89)	2139 (19.75)	425 (3.92)
Missing	7734	1707 (22.07)	1395 (18.04)	446 (5.77)
Weight categories				
Low weight category	22 140	4651 (21.01)	3960 (17.89)	1055 (4.77)
Normal weight category	37 240	7844 (21.06)	6870 (18.45)	1516 (4.07)
High weight category	22 788	4556 (19.99)	4078 (17.90)	727 (3.19)
Ethnic group				
Asians	18 914	4945 (26.14)	4255 (22.50)	1231 (6.51)
Blacks	605	118 (19.5)	99 (16.36)	30 (4.96)
Mixed	1828	324 (17.72)	280 (15.32)	83 (4.54)
Other races	627	127 (20.26)	112 (17.86)	27 (4.31)
Whites	39 071	8135 (20.82)	7258 (18.58)	1414 (3.62)
Missing	10 496	2234 (21.28)	1952 (18.60)	407 (3.88)

Demographic data for study cohort

Caribbean, White and Black African, White and Asian, Any other Mixed background; White: Any White Background (including Welsh, English, Scottish, Northern Irish, Irish, British), Gypsy, other White background; Other ethnicities: Arab and any other ethnic group.

HWChigh weight categoryLWClow weight categoryNWCnormal weight category

#### Logistic regression

**Figure 2:**
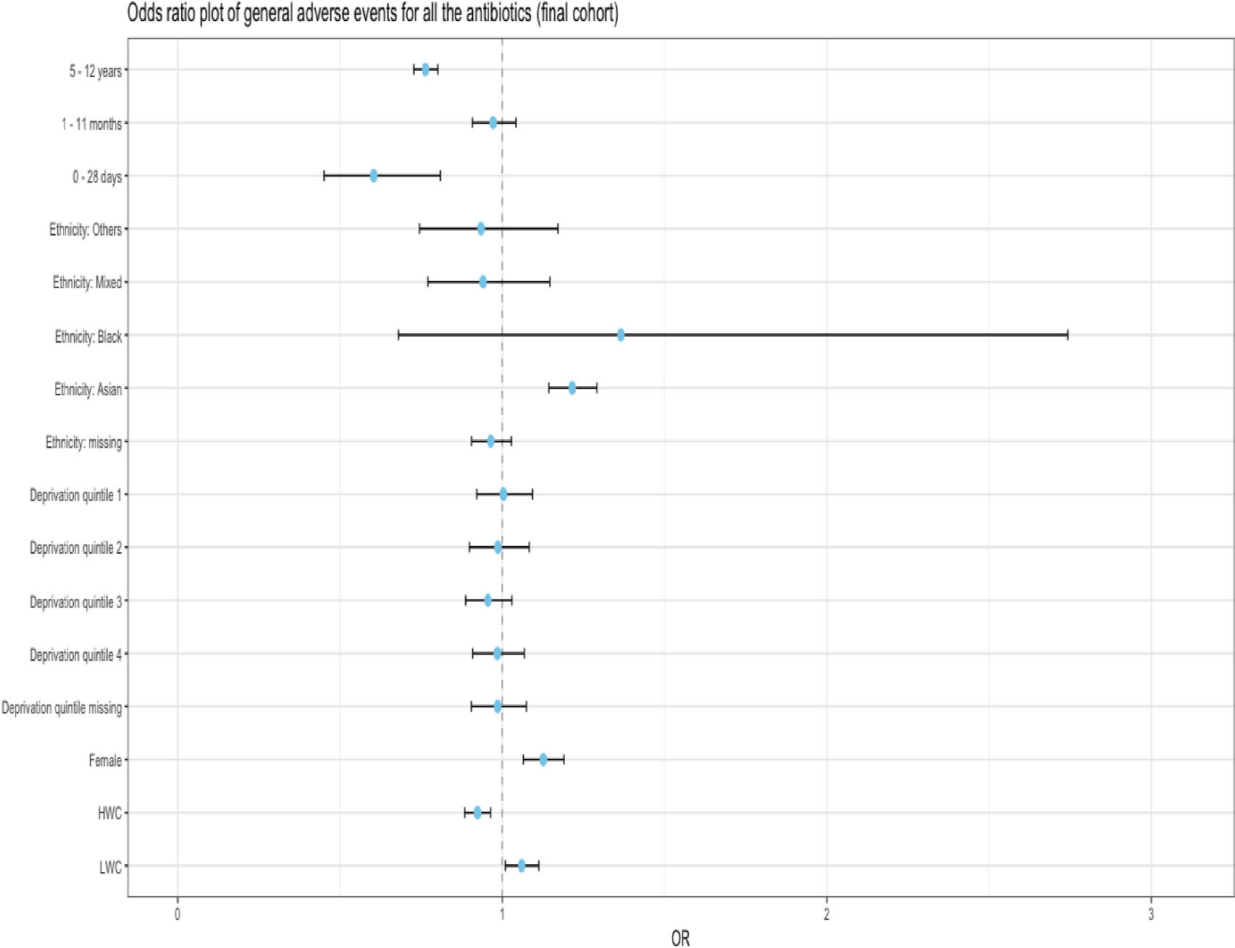
Forest plot of OR of combined adverse events after initial oral antibiotics prescriptions. The x value of 1 denotes no difference in OR between the reference group and the group being compared. Reference groups are—age band: 1–4 years, ethnicity: white, sex: male, weight category: normal weight category. HWC, high weight category; LWC, low weight category.

**Figure 3:**
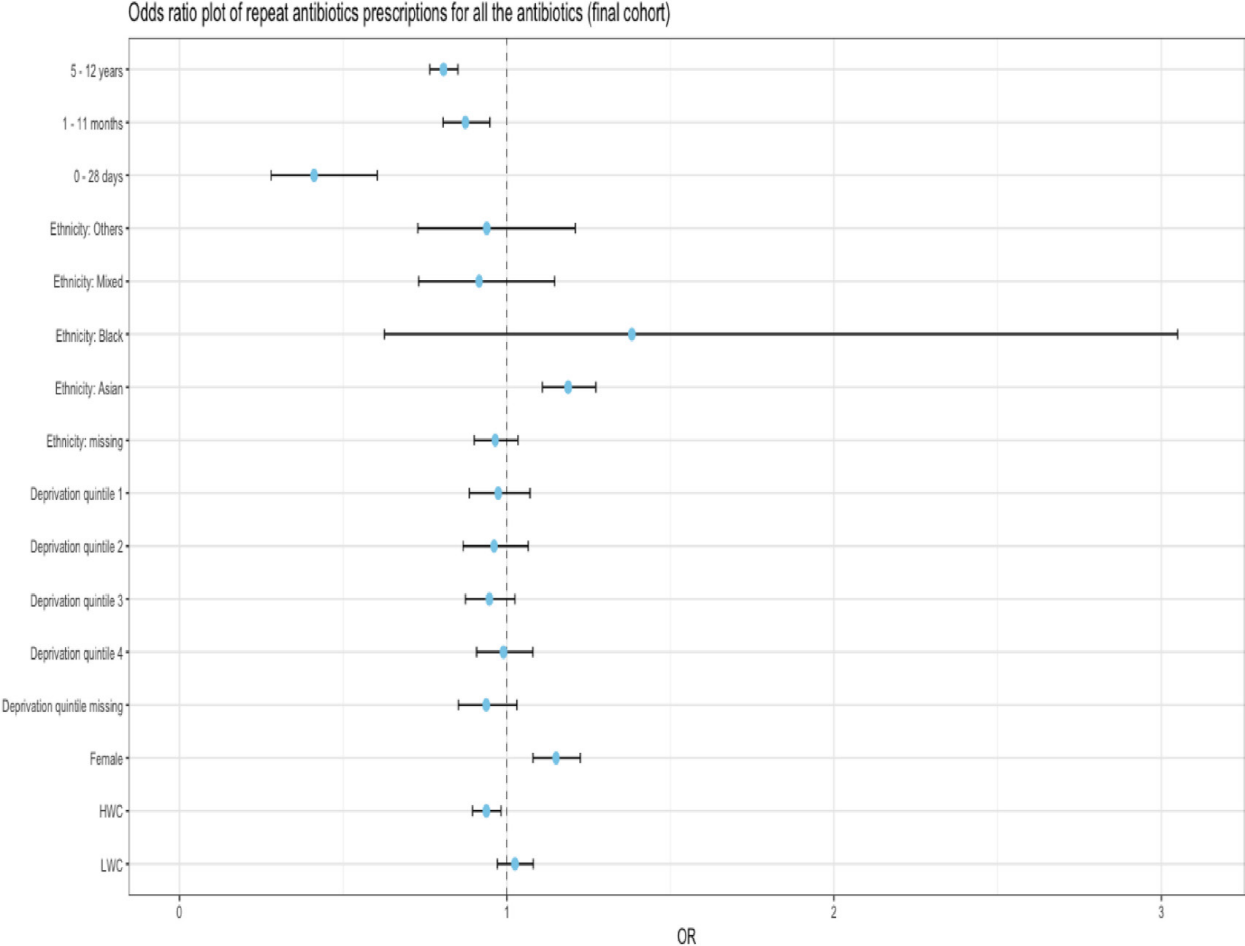
Forest plot of OR of repeat antibiotics prescription after initial oral antibiotics prescriptions. The x value of 1 denotes no difference in OR between the reference group and the group being compared. Reference groups are—age band: 1–4 years, ethnicity: white, sex: male, weight category: normal weight category. HWC, high weight category; LWC, low weight category.

**Figure 4:**
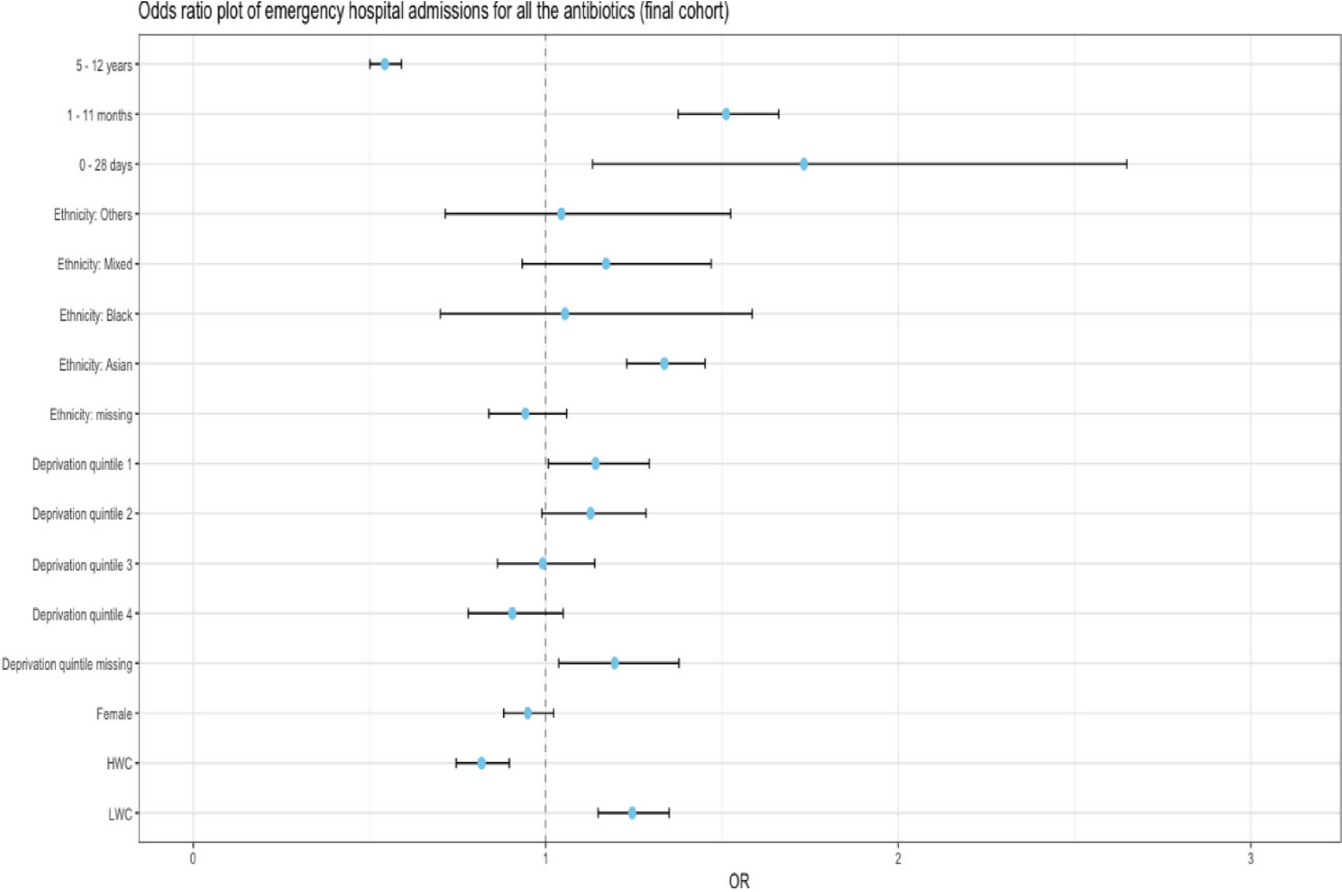
Forest plot of OR of emergency hospital admission after initial oral antibiotics prescriptions. The x value of 1 denotes no difference in OR between the reference group and the group being compared. Reference groups are—age band: 1–4 years, ethnicity: white, sex: male, weight category: normal weight category. HWC, high weight category; LWC, low weight category.

Children in the LWC had higher odds of an adverse reaction (aOR (95% CI): 1.06 (1.01, 1.11)) compared with those categorised in the NWC; while children in the HWC had lower odds 0.92 (0.88, 0.96). Females had higher odds 1.13 (1.07, 1.19) than males having adjusted for all other factors. Children in the 0–28 days and 5–12 years age bands had lower odds (0.60 (0.45, 0.81), and 0.76 (0.73, 0.81) respectively) than those in the 1–4 months age band. Asian ethnicity had higher odds than the whites (with ORs of 1.22 (1.14, 1.29)). The risk factors, ORs, upper and lower CIs can be found in online supplemental table, figure 2, figure 3 and figure 4.

## Discussion

Children who were of low weight, female or of Asian ethnic backgrounds had higher odds of adverse events following oral antibiotic prescriptions compared with their respective reference groups having adjusted for age, sex, ethnic group, deprivation quintiles and weight category. Conversely, children categorised as high weight and children in 0–28 days and 5–12 years age groups demonstrated lower odds of experiencing adverse events. Similarly, those of low weight, smaller children (aged up to 28 days or between 1 and 11 months), of Asian ethnicity, or residing in deprivation quintile 1 were found to have an increased odds of an emergency hospital admission within 5 days of the initial oral antibiotic prescribed. This was analogous to the result from investigating the repeat primary care prescription of oral antibiotics within 14 days of the initial oral antibiotic as children who were of Asian ethnicity, or female were found to have higher odds of this subset of adverse events. The reason for the observed trend is unknown and requires further investigation, ideally in a more ethnically diverse population with a more equal representation of the various age bands.

Our findings align with Bielicki *et al*’s assertion that weight, in addition to age bands, is a crucial variable in antibiotic prescription for children.^8^ Specifically, our results indicate that children classified as low weight for their sex and age band exhibit elevated odds of adverse events, consistent with existing literature.^31^ Conversely, our observation that HWC children have lower odds of adverse events compared with those of normal weight provides further support to this notion. Taken together, these findings underscore the importance of considering weight alongside age when prescribing oral antibiotics to children, offering a potential avenue to mitigate adverse events in this population.

Studies have shown that babies of Asian (Indian, Pakistani, Bangladeshi, Chinese and other Asian ethnic groups) ethnicity tend to have lower body weights in comparison to those of Caucasian ancestry.^32 33^ This observation may suggest that the increased odds of general adverse events among minority ethnic groups could be attributed, at least in part, to the lower birth weight prevalent in these populations.^34^ Children of other ethnicities show a tendency towards very high odds (OR 1.84 (95% CI 1.53, 2.19)) of adverse events. However, the prevalence of this ethnic group in Wales is small (0.86%) and results in a wide CI so the likely OR is inconclusive and would require further investigation.

Sex also appears to be associated with general adverse event outcome in children prescribed with oral antibiotics; with our result suggesting that females have higher odds than males to experience a general adverse event. Given that boys tend to have a higher weight trajectory than girls^35^; and, there is no difference in dosage based on sex, the observed increase in odds is likely linked to the weight difference between the sexes. This would further emphasise the need to prioritise weight measurement when prescribing oral antibiotics to children.

### Strengths and limitations

This study was carried out by linking routinely collected data for the whole population of Wales over a period of 10 years. It provides a valuable resource to help inform policy aimed at improving paediatric health outcomes and preventing the incidences of adverse events. Important patient demographics such as sex, deprivation quintiles, age group and weight have been investigated to help healthcare professionals improve individualised care for children in need of oral antibiotics.

Two major limitations were identified in this study. First, a formal causality assessment was not conducted.^36^ A significant challenge in pharmacovigilance is accurately pinpointing the root cause of adverse reactions to specific drugs.^37^ Despite implementing rigorous measures to establish a clear link between observed adverse reactions and the prescribed oral antibiotic, the absence of formal causality assessment limits the strength of our conclusions. Second, the study suffered from inadequate representation of minority ethnic groups in Wales,^38^ which hindered a comprehensive assessment of ethnicity’s impact on the measured outcome. Addressing these limitations in future research endeavours is crucial to enhance the robustness and generalisability of findings.

This study lays the groundwork for understanding the importance of weight measurement in the prescription of oral antibiotics. While a detailed exploration of the correlation between risk factors and adverse events necessitates focusing on specific classes of antibiotics and their indications, future research examining individual oral antibiotics can offer further insights to inform healthcare policies and enhance patient care.

## Conclusion

Our study sheds light on the significant role of weight as a crucial variable in determining adverse events following oral antibiotic prescriptions in children. Our findings highlight that children who are of low weight, female, or, of certain minority ethnic backgrounds are at heightened risk of adverse events. Conversely, children categorised as high weight and older children demonstrate lower odds of experiencing adverse events. These results underscore the importance of considering weight alongside other demographic factors when prescribing oral antibiotics to children in primary care. By prioritising weight measurement, healthcare providers can better tailor antibiotic prescriptions, potentially mitigating adverse drug reactions and improving outcomes for paediatric patients.

This finding does not overlook the fact that weight may serve as a proxy for various underlying conditions and factors that can predispose children to adverse outcomes following oral antibiotic prescriptions. While weight itself may not be the direct issue, it signifies potential links with factors such as malnutrition, intrauterine growth restriction, neglect, prematurity, immunocompromise and other health conditions. By disregarding weight and dosing-based solely on averages, we overlook the complexities of individual health profiles and miss opportunities to tailor treatments accordingly. Weight, as a measure of growth and development, is integral to monitoring overall health status. Our study underscores the importance of recognising weight as more than just a number—it represents a critical aspect of a child’s health that warrants careful consideration in antibiotic prescription practices to optimise outcomes and mitigate adverse events.

## supplementary material

10.1136/bmjpo-2024-002831 online supplemental file 1

10.1136/bmjpo-2024-002831 online supplemental file 2

10.1136/bmjpo-2024-002831 online supplemental file 3

10.1136/bmjpo-2024-002831 online supplemental file 4

10.1136/bmjpo-2024-002831 online supplemental file 5

10.1136/bmjpo-2024-002831 online supplemental file 6

## Data Availability

All data relevant to the study are included in the article or uploaded as supplementary information.
