# Supplementary material for: Association of child weight and adverse outcomes following antibiotic prescriptions in children: a national data study in Wales, UK
Source: BMJ Paediatr Open. 2024 Nov 28;8(1):e002831. doi: 10.1136/bmjpo-2024-002831 (PMC11605826; doi:10.1136/bmjpo-2024-002831)
Supplement: online supplemental file 1 [file bmjpo-8-1-s001.pdf]

APPENDIX 1: Read codes for the oral antibiotics

Read codes	Description
e15..	PHENOXYMETHYLPENICILLIN
e151.	PHENOXYMETHYLPENICILLIN 250mg capsules
e152.	PHENOXYMETHYLPENICILLIN 125mg capsules
e153.	PHENOXYMETHYLPENICILLIN 250mg tablets
e154.	PHENOXYMETHYLPENICILLIN 62.5mg/5mL syrup
e155.	PHENOXYMETHYLPENICILLIN 125mg/5mL syrup
e156.	PHENOXYMETHYLPENICILLIN granules 125mg/sachet
e157.	PHENOXYMETHYLPENICILLIN 250mg/5mL syrup
e158.	*APSIN VK 250mg tablets
e159.	*APSIN VK 125mg/5mL syrup
e15A.	PHENOXYMETHYLPENICILLIN 125mg tablets
e15B.	*RIMAPEN 250mg tablets
e15a.	APSIN VK 250mg/5mL syrup
e15b.	*CRYSTAPEN V 125mg/5mL syrup
e15c.	*CRYSTAPEN V 250mg/5mL syrup
e15d.	*DISTAQUAINE V-K 125mg tablets
e15e.	*DISTAQUAINE V-K 250mg tablets
e15f.	DISTAQUAINE V-K 62.5mg/5mL syrup
e15g.	DISTAQUAINE V-K 125mg/5mL syrup
e15h.	*DISTAQUAINE 250mg/5mL syrup
e15i.	*ECONOCIL VK 250mg capsules
e15j.	*ECONOCIL VK 125mg tablets
e15k.	*ECONOCIL VK 250mg tablets
e15l.	*STABILLIN V-K 250mg tablets
e15m.	STABILLIN V-K 62.5mg/5mL syrup
e15n.	*STABILLIN V-K 125mg/5mL syrup
e15o.	*STABILLIN V-K 250mg/5mL syrup
e15p.	*V-CIL-K 250mg capsules
e15q.	*V-CIL-K 125mg tablets
e15r.	*V-CIL-K 250mg tablets
e15s.	V-CIL-K PAEDIATRIC 62.5mg/5mL syrup
e15t.	V-CIL-K PAEDIATRIC 125mg/5mL syrup
e15u.	*V-CIL-K 250mg/5mL syrup
e15v.	*TENKICIN 250mg tablets
e15w.	PHENOXYMETHYLPENICILLIN 125mg/5mL s/f oral solution
e15x.	PHENOXYMETHYLPENICILLIN 250mg/5mL s/f oral solution
e221.	FLUCLOXACILLIN 250mg capsules
e222.	FLUCLOXACILLIN 500mg capsules
e223.	FLOXAPEN 250mg capsules
e224.	FLOXAPEN 500mg capsules
e225.	FLOXAPEN 125mg/5mL syrup
e226.	FLOXAPEN FORTE 250mg/5mL syrup
e22A.	FLUCLOXIN 125mg/5mL oral suspension
e22B.	FLUCLOXACILLIN 250mg/5mL oral suspension
e22C.	FLUCLOXACILLIN 125mg/5mL s/f oral solution
e22D.	FLUCLOXACILLIN 250mg/5mL s/f oral solution
e22a.	*LADROPEN 250mg capsules
e22b.	*LADROPEN 500mg capsules
e22c.	*STAFOXIL 250mg capsules

e22d.	*STAFOXIL 500mg capsules
e22e.	*STAPHLIPEN 250mg capsules
e22f.	*STAPHLIPEN 500mg capsules
e22j.	FLUCLOXACILLIN 125mg/5mL syrup
e22k.	FLUCLOXACILLIN 250mg/5mL syrup
e22l.	*FLUCLOMIX 250mg capsules
e22m.	FLUCLOMIX 500mg capsules
e22n.	LADROPEN 125mg/5mL suspension 100mL
e22t.	*GALFLOXIN 250mg capsules
e22u.	*GALFLOXIN 500mg capsules
e22v.	FLUCLOXACILLIN 125mg/5mL oral suspension
e22w.	*ZOXIN 250 capsules
e22x.	*ZOXIN 500 capsules
e22y.	*FLUCLOXIN 250mg capsules
e22z.	*FLUCLOXIN 500mg capsules
e311.	AMOXICILLIN 250mg capsules
e312.	AMOXICILLIN 500mg capsules
e313.	*AMOXIDIN 250mg capsules
e314.	*AMOXIDIN 500mg capsules
e315.	AMOXIL 250mg capsules
e316.	AMOXIL 500mg capsules
e317.	AMOXIL 500mg dispersible tablets
e318.	*AMOXIL 125mg/5mL syrup
e319.	*AMOXIL SF 125mg/5mL syrup
e31A.	*AMIX 125mg/5mL suspension
e31B.	*AMIX 250mg/5mL suspension
e31C.	*AMRIT 125mg/5mL suspension
e31D.	*AMRIT 250mg/5mL suspension
e31E.	*AMRIT 250mg capsules
e31F.	*AMRIT 500mg capsules
e31G.	*AMOPEN 250mg capsules
e31H.	*AMOPEN 500mg capsules
e31I.	*AMOPEN 125mg/5mL suspension
e31J.	*AMOPEN 250mg/5mL suspension
e31K.	FLEMOXIN SOLUTAB 375mg dispersible tablets
e31L.	FLEMOXIN SOLUTAB 750mg dispersible tablets
e31M.	AMOXIL FIZTAB 125mg chewable tablets
e31N.	AMOXIL FIZTAB 250mg chewable tablets
e31O.	AMOXIL FIZTAB 500mg chewable tablets
e31P.	AUGMENTIN 250/62 in 5mL suspension
e31Q.	CO-AMOXICLAV 125/31mg in 5mL suspension
e31R.	*AMOXYMED 250mg capsules
e31S.	*AMOXYMED 125mg/5mL syrup
e31T.	AUGMENTIN 625mg tablets
e31U.	CO-AMOXICLAV 625mg tablets
e31V.	ALMODAN 125mg/5mL sugar free syrup
e31W.	ALMODAN 250mg/5mL sugar free syrup
e31X.	CO-AMOXICLAV 400/57mg in 5mL sugar free suspension
e31Y.	AUGMENTIN-DUO 400/57 in 5mL sugar free suspension
e31a.	*AMOXIL SF 250mg/5mL syrup
e31b.	AMOXIL 125mg/1.25mL paediatric suspension
e31c.	*AMOXIL SF 750mg sachets

e31d.	*AMOXIL SF 3g sachets
e31h.	AUGMENTIN 375mg tablets
e31i.	AUGMENTIN 375mg dispersible tablets
e31j.	AUGMENTIN JUNIOR 125/62 in 5mL suspension
e31k.	AUGMENTIN 125/31 in 5mL paediatric suspension
e31n.	*ALMODAN 250mg capsules
e31o.	*ALMODAN 500mg capsules
e31p.	*ALMODAN 125mg/5mL syrup
e31q.	*ALMODAN 250mg/5mL syrup
e31t.	CO-AMOXICLAV 375mg tablets
e31u.	CO-AMOXICLAV 375mg dispersible tablets
e31v.	CO-AMOXICLAV 125mg/5mL suspension
e31w.	CO-AMOXICLAV 125mg/mL suspension
e31z.	CO-AMOXICLAV 250/62 in 5mL suspension
e321.	AMPICILLIN 250mg capsules
e322.	AMPICILLIN 500mg capsules
e323.	AMPICILLIN 125mg/5mL mixture
e324.	AMPICILLIN 250mg/5mL mixture
e325.	*AMFIPEN 250mg capsules
e326.	*AMFIPEN 500mg capsules
e327.	*AMFIPEN 125mg/5mL syrup
e328.	*AMFIPEN 250mg/5mL syrup
e329.	*AMFIPEN 250mg injection
e32A.	*RIMACILLIN 250mg capsules
e32B.	*RIMACILLIN 500mg capsules
e32C.	*RIMACILLIN 125mg/5mL syrup
e32D.	*RIMACILLIN 250mg/5mL syrup
e32E.	*AMPICILLIN 250mg injection
e32F.	AMPICILLIN 500mg injection
e32G.	AMPICILLIN 125mg/1.25mL paediatric suspension
e32H.	AMPICILLIN 125mg/5mL sugar free suspension
e32J.	AMPICILLIN 250mg/5mL sugar free suspension
e32K.	Ampicillin 125mg/5mL oral suspension
e32b.	*AMPILAR 250mg capsules
e32c.	*AMPILAR 500mg capsules
e32d.	*AMPILAR 125mg/5mL syrup
e32e.	*AMPILAR 250mg/5mL syrup
e32f.	*BRITCIN 250mg capsules
e32g.	*BRITCIN 500mg capsules
e32h.	PENBRITIN 250mg capsules
e32i.	PENBRITIN 500mg capsules
e32j.	*PENBRITIN 125mg tablets
e32k.	PENBRITIN 125mg/5mL syrup
e32l.	PENBRITIN 250mg/5mL syrup
e32m.	PENBRITIN 100mg/mL paediatric suspension
e32p.	*VIDOPEN 250mg capsules
e32q.	*VIDOPEN 500mg capsules
e32r.	*VIDOPEN 125mg/5mL syrup
e32s.	*VIDOPEN 250mg/5mL syrup
e32v.	*AMPITRIN 250mg capsules
e32w.	*AMPITRIN 500mg capsules
e32x.	AMPITRIN 125mg/5mL oral suspension
e32y.	AMPITRIN 250mg/5mL oral suspension
e32z.	*AMPICILLIN 125mg tablets

e334.	*FLU-AMP 250/250mg capsules
e335.	MAGNAPEN 500mg capsules
e336.	*MAGNAPEN 250mg/5mL syrup
e339.	CO-FLUAMPICIL 250mg/250mg capsules
e33a.	CO-FLUAMPICIL 125/125mg syrup
e33h.	*UNASYN 375mg tablets
e33i.	*SULTAMICILLIN 375mg tablets
e3A..	AMOXICILLIN [2]
e3A1.	*RESPILLIN 250mg capsules
e3A2.	*RESPILLIN 500mg capsules
e3A3.	RESPILLIN 125mg/5mL oral suspension
e3A4.	RESPILLIN 250mg/5mL oral suspension
e3A5.	RESPILLIN 125mg/5mL sugar free suspension
e3A6.	RESPILLIN 250mg/5mL sugar free suspension
e3A7.	*AMICLAV 250mg/125mg tablets
e3A8.	*RANCLAV 375mg tablets
e3A9.	*RANCLAV 625mg tablets
e3AA.	RANCLAV 125mg/31mg sugar free suspension
e3AB.	RANCLAV 250mg/62mg sugar free suspension
e3z..	AMOXICILLIN [GENERIC ADDITIONS]
e3z1.	*AMORAM 250mg capsules
e3z2.	*AMORAM 500mg capsules
e3z3.	*AMORAM 125mg/5mL suspension
e3z4.	*AMORAM 250mg/5mL suspension
e3z5.	AMIX 250mg capsules
e3z6.	AMIX 500mg capsules
e3z7.	*GALENAMOX 250mg capsules
e3z8.	*GALENAMOX 500mg capsules
e3z9.	GALENAMOX 125mg/5mL suspension
e3zA.	GALENAMOX TP 250mg capsules
e3zB.	GALENAMOX TP 500mg capsules
e3zC.	*ZOXYCIL 250 capsules
e3zD.	*ZOXYCIL 500 capsules
e3zE.	AMOXICILLIN 125mg/sachet sugar free powder
e3zF.	AMOXIDENT 250mg capsules
e3zG.	AMOXIDENT 500mg capsules
e3za.	GALENAMOX 250mg/5mL suspension
e3zb.	GALENAMOX 125mg/5mL sugar free suspension
e3zc.	GALENAMOX 250mg/5mL sugar free suspension
e3zf.	*RIMOXALLIN 125mg/5mL syrup
e3zg.	*RIMOXALLIN 250mg capsules
e3zh.	*RIMOXALLIN 500mg capsules
e3zj.	*RIMOXALLIN 250mg/5mL syrup
e3zk.	AMOXICILLIN 125mg/5mL sugar free suspension
e3zl.	AMOXYCILLIN 500mg dispersible tablets
e3zm.	AMOXICILLIN 125mg/5mL syrup
e3zn.	AMOXICILLIN 250mg/5mL syrup
e3zo.	AMOXICILLIN 125mg/1.25mL paediatric suspension
e3zp.	AMOXYCILLIN powder 750mg/sachet
e3zq.	AMOXICILLIN powder 3g/sachet
e3zu.	AMOXICILLIN 250mg/5mL sugar free suspension
e3zv.	AMOXYCILLIN 125mg s/f chewable tablets
e3zw.	AMOXYCILLIN 250mg s/f chewable tablets
e3zx.	AMOXYCILLIN 500mg s/f chewable tablets

e3zy.	AMOXYCILLIN 375mg s/f dispersible tablets
e3zz.	AMOXYCILLIN 750mg s/f dispersible tablets
e52..	PIVMECILLINAM HYDROCHLORIDE
e521.	SELEXID 200mg tablets
e522.	SELEXID 100mg/sachet suspension
e52v.	PIVMECILLINAM 100mg/sachet suspension
e52w.	PIVMECILLINAM HYDROCHLORIDE 200mg tablets
e69..	CEFALEXIN
e691.	CEFALEXIN 250mg capsules
e692.	CEFALEXIN 500mg capsules
e693.	CEFALEXIN 250mg tablets
e694.	CEFALEXIN 500mg tablets
e695.	CEFALEXIN 125mg/5mL mixture
e696.	CEFALEXIN 250mg/5mL mixture
e697.	CEFALEXIN 500mg/5mL syrup
e698.	CEPOREX 250mg capsules
e699.	CEPOREX 500mg capsules
e69A.	*TENKOREX 250mg capsules
e69B.	*TENKOREX 500mg capsules
e69C.	*TENKOREX 125mg/5mL suspension
e69D.	*TENKOREX 250mg/5mL suspension
e69E.	*TENKOREX 500mg tablets
e69F.	*KIFLONE 500mg tablets
e69G.	*KIFLONE 250mg capsules
e69H.	*KIFLONE 500mg capsules
e69J.	*KIFLONE 125mg/5mL syrup
e69K.	*KIFLONE 250mg/5mL syrup
e69a.	CEPOREX 250mg tablets
e69b.	CEPOREX 500mg tablets
e69c.	CEPOREX 125mg/1.25mL paediatric drops
e69d.	*CEPOREX 125mg/5mL suspension
e69e.	*CEPOREX 250mg/5mL suspension
e69f.	CEPOREX 125mg/5mL syrup
e69g.	CEPOREX 250mg/5mL syrup
e69h.	CEPOREX 500mg/5mL syrup
e69i.	KEFLEX 250mg capsules
e69j.	KEFLEX 500mg capsules
e69k.	KEFLEX 250mg tablets
e69l.	KEFLEX 500mg tablets
e69m.	KEFLEX 125mg/5mL suspension
e69n.	KEFLEX 250mg/5mL suspension
e69o.	KEFLEX-C 125mg chewable tablets
e69p.	KEFLEX-C 250mg chewable tablets
e69q.	*CEPOREX 1g tablets
e69v.	CEFALEXIN 125mg/5mL syrup
e69w.	CEFALEXIN 250mg/5mL syrup
e69x.	*CEPHALEXIN 1g tablets
e69y.	CEPHALEXIN 125mg/1.25mL paediatric drops
e61..	CEFACLOR
e611.	*DISTACLOR 250mg capsules
e612.	DISTACLOR 125mg/5mL suspension
e613.	DISTACLOR 250mg/5mL suspension
e614.	CEFACLOR 250mg capsules
e615.	CEFACLOR 125mg/5mL suspension

e616.	CEFACLOR 250mg/5mL suspension
e617.	DISTACLOR 500mg capsules
e618.	CEFACLOR 500mg capsules
e619.	DISTACLOR MR 375mg m/r tablets
e61A.	KEFTID 250mg capsules
e61B.	KEFTID 500mg capsules
e61C.	CEFACLOR 125mg/5mL sugar free suspension
e61D.	CEFACLOR 250mg/5mL sugar free suspension
e61E.	KEFTID 125mg/5mL sugar free suspension
e61F.	KEFTID 250mg/5mL sugar free suspension
e61G.	BACTICLOR MR 375mg m/r tablets
e61a.	CEFACLOR 375mg m/r tablets
e61b.	DISTACLOR MR 500mg m/r tablets
e61c.	*CEFACLOR 500mg m/r tablets
e62..	CEFADROXIL
e621.	*BAXAN 500mg capsules
e622.	*BAXAN 125mg/5mL suspension
e623.	*BAXAN 250mg/5mL suspension
e624.	*BAXAN 500mg/5mL suspension
e625.	CEFADROXIL 125mg/5mL suspension
e626.	CEFADROXIL 250mg/5mL suspension
e627.	CEFADROXIL 500mg capsules
e62w.	*CEFADROXIL 500mg capsules
e62x.	*CEFADROXIL 500mg capsules
e62z.	CEFADROXIL 500mg/5mL suspension
e684.	ZINNAT 125mg tablets
e685.	ZINNAT 250mg tablets
e686.	CEFUROXIME 125mg tablets
e687.	CEFUROXIME 250mg tablets
e689.	ZINNAT 125mg/5mL suspension
e68a.	CEFUROXIME 125mg/5mL suspension
e68b.	ZINNAT 125mg/sachet suspension
e68c.	CEFUROXIME 125mg/sach for suspension
e6h..	CEFIXIME
e6h1.	CEFIXIME 200mg tablets
e6h2.	CEFIXIME 100mg/5mL suspension
e6h3.	SUPRAX 200mg tablets
e6h4.	SUPRAX 100mg/5mL paediatric suspension 37.5mL
e6h5.	SUPRAX 100mg/5mL paediatric suspension 75mL
e6h6.	SUPRAX 100mg/5mL paediatric suspension 50mL
e6h7.	SUPRAX 100mg/5mL paediatric suspension 100mL
e911.	ERYTHROMYCIN 250mg e/c tablets
e912.	ERYTHROMYCIN 500mg tablets
e913.	ERYTHROMYCIN STEARATE 250mg tablets
e914.	ERYTHROMYCIN STEARATE 500mg tablets
e915.	ARPIMYCIN 125mg/5mL sugar free suspension
e916.	ARPIMYCIN 250mg/5mL sugar free suspension
e917.	ARPIMYCIN 500mg/5mL sugar free suspension
e918.	*ERYCEN 250mg tablets
e919.	*ERYCEN 500mg tablets
e91A.	ERYTHROPED FORTE granules 500mg/sachet
e91B.	ERYTHROPED P.I. granules 125mg/sachet
e91C.	ERYTHROPED P.I. 125mg/5mL sugar free suspension 140mL

e91D.	ERYTHROPED 250mg/5mL sugar free suspension 140mL
e91E.	ERYTHROMYCIN 125mg/5mL sugar free suspension
e91F.	ERYTHROMYCIN 250mg/5mL sugar free suspension
e91G.	*ROMMIX-125 suspension
e91H.	*ROMMIX-250 tablets
e91I.	KERYMAX 250mg e/c granules in capsules
e91J.	*ROMMIX-500 tablets
e91L.	*ERYTHROMYCIN 250mg capsules
e91M.	ERYTHROMYCIN 125mg/sachet granules
e91N.	ERYTHROMYCIN 250mg/sachet granules
e91P.	ERYTHROMYCIN 500mg/sachet granules
e91Q.	ERYTHROMYCIN 1g/sachet granules
e91R.	ERYTHROMYCIN 500mg/5mL sugar free suspension
e91S.	TILORYTH 250mg e/c granules in capsules
e91T.	ERYMIN 250mg/5mL sugar free suspension
e91U.	ARPIMYCIN 125mg/5mL suspension
e91V.	ARPIMYCIN 250mg/5mL suspension
e91W.	ARPIMYCIN 500mg/5mL suspension
e91X.	ERYTHROMYCIN 250mg e/c granules in capsules
e91Y.	ERYTHROPED FORTE SF 500mg/5mL sugar free suspension
e91Z.	PRIMACINE 125mg/5mL suspension 100mL
e91a.	ERYMAX 250mg e/c granules in capsules
e91b.	ERYTHROCIN 250mg tablets
e91c.	ERYTHROCIN 500mg tablets
e91e.	*ERYTHROLAR 250mg tablets
e91f.	*ERYTHROLAR 500mg tablets
e91g.	ERYTHROLAR 250mg/5mL suspension
e91h.	*ERYTHROMID 250mg tablets
e91i.	*ERYTHROMID DS 500mg tablets
e91j.	ERYTHROPED P.I. 125mg/5mL suspension
e91k.	ERYTHROPED 250mg/5mL suspension 140mL
e91l.	ERYTHROPED 250mg/sachet sugar free granules
e91m.	ERYTHROPED FORTE 500mg/5mL suspension
e91n.	ERYTHROPED A 500mg tablets
e91o.	*ILOSONE 250mg capsules
e91p.	*ILOSONE 500mg tablets
e91q.	*ILOSONE 125mg/5mL suspension
e91r.	ILOSONE FORTE 250mg/5mL suspension
e91s.	*ILOTYCIN 250mg tablets
e91t.	RETCIN 250mg tablets
e91u.	ERYTHROMYCIN 125mg/5mL suspension
e91v.	ERYTHROMYCIN 250mg/5mL suspension
e91w.	ERYTHROMYCIN 500mg/5mL suspension
e91x.	ERYTHROPED A 1g/sachet granules
e91y.	ERYMAX SPRINKLE 125mg capsules
e91z.	ERYTHROPED 250mg/sachet granules
e921.	CLARITHROMYCIN 250mg tablets
e922.	KLARICID 250mg tablets 14CP
e923.	CLARITHROMYCIN 125mg/5mL paediatric suspension
e924.	KLARICID 125mg/5mL paediatric suspension
e927.	CLARITHROMYCIN 500mg tablets
e928.	KLARICID 500mg tablets

e929.	CLARITHROMYCIN 500mg m/r tablets
e92A.	KLARICID XL 500mg m/r tablets
e92B.	CLARITHROMYCIN 250mg/sachet granules
e92C.	KLARICID adult 250mg/sachet granules
e92D.	CLARITHROMYCIN 250mg/5mL paediatric suspension
e92E.	KLARICID 250mg/5mL paediatric suspension
e92F.	CLARITHROMYCIN 125mg granules straw
e92G.	*CLAROSIP 125mg granules straw
e92H.	CLARITHROMYCIN 187.5mg granules straw
e92I.	CLAROSIP 187.5mg granules straw
e92J.	CLARITHROMYCIN 250mg granules straw
e92K.	CLAROSIP 250mg granules straw
e92L.	XETININ XL 500mg m/r/ tablets
e92M.	FEBZIN XL 500mg m/r tablets
e92N.	MYCIFOR XL 500mg m/r tablets
e931.	AZITHROMYCIN 250mg capsules
e932.	AZITHROMYCIN 40mg/mL suspension
e933.	ZITHROMAX 250mg capsules
e934.	ZITHROMAX 40mg/mL suspension 15mL
e935.	ZITHROMAX 40mg/mL suspension 22.5mL
e936.	ZITHROMAX 40mg/mL suspension 30mL
e937.	AZITHROMYCIN 500mg tablets
e938.	*ZITHROMAX 500mg tablets
e939.	CLAMELLE AZITHROMYCIN 500mg tablets
e95..	ERYTHROMYCIN [2]
e951.	PRIMACINE 125mg/5mL suspension 140mL
e952.	PRIMACINE 250mg/5mL suspension 100mL
e953.	PRIMACINE 250mg/5mL suspension 140mL
e954.	PRIMACINE 500mg/5mL suspension 100mL
e955.	PRIMACINE 500mg/5mL suspension 140mL
ea11.	DALACIN C 75mg capsules
ea12.	DALACIN C 150mg capsules
ea13.	DALACIN C 75mg/5mL paediatric suspension
ea1v.	CLINDAMYCIN 75mg capsules
ea1w.	CLINDAMYCIN 150mg capsules
ea1x.	*CLINDAMYCIN 75mg/5mL syrup
ec11.	CO-TRIMOXAZOLE 480mg tablets
ec12.	CO-TRIMOXAZOLE 480mg dispersible tablets
ec13.	CO-TRIMOXAZOLE 960mg tablets
ec14.	CO-TRIMOXAZOLE 960mg dispersible tablets
ec15.	CO-TRIMOXAZOLE 120mg tablets
ec16.	CO-TRIMOXAZOLE 480mg/5mL mixture
ec17.	CO-TRIMOXAZOLE 240mg/5mL mixture
ec1A.	CO-TRIMOXAZOLE 240mg/5mL sugar free suspension
ec1B.	CO_TRIMOXAZOLE 480mg/5mL suspension
ec21.	*BACTRIM 480mg tablets
ec22.	BACTRIM 480mg dispersible tablets
ec23.	BACTRIM 960mg double strength tablets
ec24.	BACTRIM PAEDIATRIC 120mg tablets
ec25.	*BACTRIM 480mg/5mL suspension
ec26.	BACTRIM 240mg/5mL paediatric syrup
ec29.	CHEMOTRIM 240mg/5mL suspension

ec2a.	*COMOX 480mg tablets
ec2b.	COMOX 480mg dispersible tablets
ec2c.	*COMOX FORTE 960mg tablets
ec2d.	COMOX 240mg/5mL paediatric suspension
ec2e.	FECTRIM STANDARD 480mg dispersible tablets
ec2f.	FECTRIM FORTE 960mg dispersible tablets
ec2g.	FECTRIM 120mg paediatric tablets
ec2h.	*LARATRIM 480mg tablets
ec2i.	*LARATRIM FORTE 960mg tablets
ec2j.	*LARATRIM 480mg/5mL suspension
ec2k.	LARATRIM 240mg/5mL paediatric suspension
ec2l.	SEPTRIN 480mg tablets
ec2m.	SEPTRIN 480mg dispersible tablets
ec2n.	SEPTRIN FORTE 960mg tablets
ec2o.	SEPTRIN PAEDIATRIC 120mg dispersible tablets
ec2p.	SEPTRIN 480mg/5mL adult suspension
ec2q.	SEPTRIN 240mg/5mL paediatric suspension
ec2t.	*COMIXCO 80/400 tablets
ec2u.	*COMIXCO 160/800 tablets
ec2v.	COMIXCO 40/200/5mL paediatric suspension
ec2w.	COMIXCO 80/400 dispersible tablets
ecc1.	TRIMETHOPRIM 100mg tablets
ecc2.	TRIMETHOPRIM 200mg tablets
ecc3.	*TRIMETHOPRIM 300mg tablets
ecc4.	TRIMETHOPRIM 50mg/5mL sugar free suspension
ecc6.	*IPRAL 100mg tablets
ecc7.	*IPRAL 200mg tablets
ecc8.	IPRAL SF 50mg/5mL paediatric suspension
ecc9.	*MONOTRIM 100mg tablets
ecca.	*MONOTRIM 200mg tablets
eccb.	MONOTRIM 50mg/5mL sugar free suspension
eccd.	*SYRAPRIM 100mg tablets
ecce.	*SYRAPRIM 300mg tablets
eccf.	*SYRAPRIM 100mg/5mL injection
eccg.	TIEMPE 100mg tablets
ecch.	TIEMPE 200mg tablets
ecci.	*TRIMOGAL 100mg tablets
eccj.	*TRIMOGAL 200mg tablets
ecck.	*TRIMOPAN 100mg tablets
eccl.	*TRIMOPAN 200mg tablets
eccm.	TRIMOPAN 50mg/5mL sugar free suspension
eccn.	*TRIPRIMIX 200mg tablets
ef11.	METRONIDAZOLE 200mg tablets
ef12.	METRONIDAZOLE 400mg tablets
ef1A.	METRONIDAZOLE 200mg/5mL suspension
ef1D.	METRONIDAZOLE 500mg tablets
ef1c.	FLAGYL 200mg tablets
ef1d.	FLAGYL 400mg tablets
ef1g.	FLAGYL S suspension 100mL
ef1l.	*METROLYL 200mg tablets
ef1m.	*METROLYL 400mg tablets
ef1r.	*NIDAZOL 200mg tablets
ef1s.	VAGINYL 200mg tablets
ef1t.	VAGINYL 400mg tablets

ef1u.	*ZADSTAT 200mg tablets
eg1..	NITROFURANTOIN
eg11.	NITROFURANTOIN 50mg tablets
eg12.	NITROFURANTOIN 100mg tablets
eg13.	FURADANTIN 50mg tablets
eg14.	FURADANTIN 100mg tablets
eg15.	FURADANTIN 25mg/5mL sugar free suspension
eg16.	MACRODANTIN 50mg capsules
eg17.	MACRODANTIN 100mg capsules
eg18.	URANTOIN 50mg tablets
eg19.	URANTOIN 100mg tablets
eg1A.	MACROBID 100mg m/r capsules
eg1B.	GENFURA 50mg tablets
eg1C.	GENFURA 100mg tablets
eg1w.	NITROFURANTOIN 100mg m/r capsules
eg1x.	NITROFURANTOIN 25mg/5mL sugar free suspension
eg1y.	NITROFURANTOIN 50mg capsules
eg1z.	NITROFURANTOIN 100mg capsules
eg61.	CIPROXIN 250mg tablets
eg64.	CIPROXIN 500mg tablets
eg65.	CIPROXIN 750mg tablets
eg67.	CIPROFLOXACIN 100mg tablets
eg68.	*CIPROXIN 100mg tablets
eg69.	CIPROFLOXACIN 5g/100mL oral suspension
eg6A.	CIPROXIN 5g/100mL oral suspension
eg6v.	CIPROFLOXACIN 750mg tablets
eg6w.	CIPROFLOXACIN 500mg tablets
eg6x.	CIPROFLOXACIN 250mg tablets
