# Supplementary material for: Association of child weight and adverse outcomes following antibiotic prescriptions in children: a national data study in Wales, UK
Source: BMJ Paediatr Open. 2024 Nov 28;8(1):e002831. doi: 10.1136/bmjpo-2024-002831 (PMC11605826; doi:10.1136/bmjpo-2024-002831)
Supplement: online supplemental file 2 [file bmjpo-8-1-s002.pdf]

APPENDIX 2: Risk factors for adverse events in children prescribed oral antibiotics in the GP

Risk factors	Variable from routine data	source
Deprivation quintile	Welsh index of multiple deprivation 2014 overall index quartile.	Welsh Demographic Service Dataset (WDSD)
Ethnicity	Ethnic group description	Patient Episode Dataset for Wales (PEDW), National Community Child Health Database (NCCHD)
Sex	Gender codes	Welsh Longitudinal General Practice Dataset (WLGP) – Welsh Primary Care
Weight	Patient weight values within 30 days of oral antibiotics prescription date	WLGP
Age band	Patient age at oral antibiotics prescription date (prescription date – Week of Birth (WOB))	WLGP

Cohort selection (inclusion and exclusion criteria)

- Children born in Wales.
- Study population include children (aged 0 to 12 years) with a GP oral antibiotics prescription record (WLGP dataset)
- Weight record in WLGP.
- Weight record was within 30 days before or after oral antibiotics prescription.

Datasets used: WDSD, WLGP, PEDW, NCCHD.
