# Supplementary material for: Association of child weight and adverse outcomes following antibiotic prescriptions in children: a national data study in Wales, UK
Source: BMJ Paediatr Open. 2024 Nov 28;8(1):e002831. doi: 10.1136/bmjpo-2024-002831 (PMC11605826; doi:10.1136/bmjpo-2024-002831)
Supplement: online supplemental file 3 [file bmjpo-8-1-s003.pdf]

**Appendix 3**: Read Codes for records of adverse events in the GP

| read codes | description |
|---|---|
| SL05. | Cephalosporin group poisoning - |
| SL050 | Cefalexin poisoning |
| SL051 | Cephaloglycin poisoning |
| SL052 | Cephaloridine poisoning |
| SL053 | Cephalothin poisoning |
| SL052 | Cephalosporin poisoning NOS |
| TJ05z | Adverse reaction to cephalosporin NOS |
| T105. | Adverse reaction to cephalosporin group |
| TJ050 | Adverse reaction to cefacior |
| TJ051 | Adverse reaction to cefadroxil |
| TJ052 | Adverse reaction to cefotaxime |
| TJ053 | Adverse reaction to cefoxitin |
| TJ054 | Adverse reaction to cefsulodin sodium |
| TJ055 | Adverse reaction to ceftazidime |
| TJ056 | Adverse reaction to ceftizoxime |
| TJ057 | Adverse reaction to cephalexin |
| TJ058 | Adverse reaction to cephalothin |
| 1J059 | Adverse reaction to cephamandole |
| TJ05A | Adverse reaction to cephazolin |
| TJ05B | Adverse reaction to cephradine |
| TJ05z | Adverse reaction to cephalosporin NOS |
| U6001 | [X] Adverse reaction to cephalosporin NOS |
| Xa5ru | Macrolide allergy |
| Xa5rv | Erythromycin allergy |
| Xa5rw | Clarithromycin allergy |
| Xa5rx | Azithromycin allergy |
| Xa6Pw | Macrolide overdose |
| Xa6Px | Erythromycin overdose |
| Xa6Q1 | Azithromycin overdose |
| Xa6Q5 | Clarithromycin overdose |
| Xa5TR | Macrolide adverse reaction |
| Xa5TS | Erythromycin adverse reaction |
| Xa5TT | Clarithromycin adverse reaction |
| Xa5TU | Azithromycin adverse reaction |
| TJ03z | Adverse reaction to macrolide NOS |
| XM1Fr | Adverse reaction to macrolide group |
| TJ03. | Adverse reaction to erythromycin and other macrolides |
| TJ030 | Adverse reaction to erythromycin |
| TJ031 | Adverse reaction to oleandomycin |
| TJ032 | Adverse reaction to spiramycin |
| XE1oI | Erythromycin and macrolide poisoning |
| SL03z | Erythromycin or macrolide poisoning NOS |
| TJ03. | Adverse reaction to erythromycin and other macrolides |
| U6003 | [X]Macrolides causing adverse effects in therapeutic use |

| | |
|---|---|
| Xa5s3 | Nitrofurantoin allergy |
| 14LI. | H/O: nitrofurantoin allergy |
| Xa6QP | Nitrofuran derivative overdose |
| Xa5Ta | Nitrofurantoin adverse reaction |
| Xa56l | Accidental nitrofuran derivative poisoning |
| Xa56m | Intentional nitrofuran derivative poisoning |
| Xa56n | Nitrofuran derivative poisoning of undetermined intent |
| TJ1z2 | Adverse reaction to nitrofurantoin |
| Xa6QQ | Accidental nitrofuran derivative overdose |
| Xa6QR | Intentional nitrofuran derivative overdose |
| Xa56l | Accidental nitrofuran derivative poisoning |
| Xa56m | Intentional nitrofuran derivative poisoning |
| Xa6QS | Nitrofuran derivative overdose of undetermined intent |
| Xa56n | Nitrofuran derivative poisoning of undetermined intent |
| Xa5tS | Nitroimidazole allergy |
| Xa5tT | Metronidazole allergy |
| Xa5tV | Nimorazole allergy |
| Xa5Uz | Nitroimidazole adverse reaction |
| Xa5V0 | Metronidazole adverse reaction |
| Xa5V1 | Tinidazole adverse reaction |
| Xa5V2 | Nimorazole adverse reaction |
| SL00. | Penicillin poisoning |
| SL000 | Ampicillin poisoning |
| SL001 | Cloxacillin poisoning |
| SL002 | Carbenicillin poisoning |
| SL003 | Penicillin G poisoning |
| SL00z | Penicillin poisoning NOS |
| SL003 | Penicillin G poisoning |
| SL340 | Penicillinase poisoning |
| e1... | PENICILLINASE SENS PENICILLINS |
| e11.. | BENZYLPENICILLIN(PENICILLIN G) |
| e12.. | *BENETHAMINE PENICILLIN |
| e13.. | *BENZATHINE PENICILLIN |
| e14.. | *PHENETHICILLIN |
| e15.. | PHENOXYMETHYLPENICILLIN |
| e16.. | PROCAINE PENICILLIN |
| TJ00. | Adverse reaction to penicillins |
| TJ000 | Adverse reaction to natural penicillins |
| TJ001 | Adverse reaction to cloxacillin |
| TJ002 | Adverse reaction to flucloxacillin |
| TJ003 | Adverse reaction to amoxycillin |
| TJ004 | Adverse reaction to ampicillin |
| TJ005 | Adverse reaction to bacampicillin |
| TJ006 | Adverse reaction to ciclacillin |
| TJ007 | Adverse reaction to mezlocillin |
| TJ008 | Adverse reaction to pivampicillin |
| TJ009 | Adverse reaction to talampicillin |
| TJ009 | Adverse reaction to talampicillin |
| TJ00A | Adverse reaction to azlocillin |
| TJ00B | Adverse reaction to carbenicillin |

| | |
|---|---|
| TJ00C | Adverse reaction to carfecillin sodium |
| TJ00D | Adverse reaction to piperacillin |
| TJ00E | Adverse reaction to ticarcillin |
| TJ00F | Adverse reaction to mecillinam |
| TJ00G | Adverse reaction to pivmecillinam |
| TJ00z | Adverse reaction to penicillin NOS |
| U6000 | [X]Penicillins causing adverse effects in therapeutic use |
| Xa5s2 | Trimethoprim allergy |
| Xa6QL | Trimethoprim overdose |
| Xa6QM | Accidental trimethoprim overdose |
| Xa6QN | Intentional trimethoprim overdose |
| Xa6QO | Trimethoprim overdose of undetermined intent |
| Xa56h | Trimethoprim poisoning |
| Xa56i | Accidental trimethoprim poisoning |
| Xa56j | Intentional trimethoprim poisoning |
| Xa56k | Trimethoprim poisoning of undetermined intent |
| 14LE. | H/O: trimethoprim allergy |
| Xa5TZ | Trimethoprim adverse reaction |
| TJ0yC | Adverse reaction to trimethoprim |
| Xa6QM | Accidental trimethoprim overdose |
| Xa6QN | Intentional trimethoprim overdose |
| Xa56j | Intentional trimethoprim poisoning |
| Xa6QO | Trimethoprim overdose of undetermined intent |
| Xa56k | Trimethoprim poisoning of undetermined intent |
