# Supplementary material for: Association of child weight and adverse outcomes following antibiotic prescriptions in children: a national data study in Wales, UK
Source: BMJ Paediatr Open. 2024 Nov 28;8(1):e002831. doi: 10.1136/bmjpo-2024-002831 (PMC11605826; doi:10.1136/bmjpo-2024-002831)
Supplement: online supplemental file 4 [file bmjpo-8-1-s004.pdf]

APPENDIX 4: Adverse events data source

Adverse events	Variable from routine data	source
Patient death within 5 days	Death date	Annual District Death Extract (ADDE)
Repeat GP antibiotic prescription within 14 days	Event date, antibiotics codes	Welsh Longitudinal General Practice Dataset (WLGP)
Non-elective hospital/emergency admission within 5 days of initial prescription	Admission date	Emergency Department Dataset (EDDS),<br>Patient Episode Dataset for Wales (PEDW)
GP record of toxicity, poisoning, overdose, allergy or hypersensitivity within 14 days	Event date, event code	WLGP

These records merged (row-bind) to the main dataset and arranged chronologically to detect the adverse outcomes.

Datasets used: ADDE, WLGP, and, PEDW.
