# Supplementary material for: Association of child weight and adverse outcomes following antibiotic prescriptions in children: a national data study in Wales, UK
Source: BMJ Paediatr Open. 2024 Nov 28;8(1):e002831. doi: 10.1136/bmjpo-2024-002831 (PMC11605826; doi:10.1136/bmjpo-2024-002831)

APPENDIX 5: Information on the sensitivity group

*Table 1: Characteristics of the sensitivity group*

| | Final cohort (71,541) | | sensitivity group (310,432) | |
|---|---|---|---|---|
| Sex | | | | |
| Male | 36,762 | 50.72% | 155,847 | 50.20% |
| Female | 34,779 | 49.28% | 154,585 | 49.80% |

*Table 2: adjusted odds ratio for an adverse drug event*

| variables | OR | Lower CI | Upper CI | P values |
|---|---|---|---|---|
| Weight category | | | | |
| HWC | 0.95 | 0.92 | 0.98 | 0.265 |
| LWC | 1.03 | 0.98 | 1.08 | 0.0000 |
| NWC | - | - | - | - |
| Sex | | | | |
| Female | 1.08 | 1.04 | 1.12 | 0.000 |
| Male | - | - | - | - |
| Ethnicity | | | | |
| Asian | 1.31 | 1.26 | 1.36 | 0.000 |
| Black | 1.43 | 0.90 | 2.25 | 0.129 |
| Mixed | 0.99 | 0.90 | 1.10 | 0.908 |
| Missing | 0.99 | 0.95 | 1.03 | 0.504 |
| Other ethnicities | 1.13 | 0.86 | 1.48 | 0.389 |
| White | - | - | - | - |
| Deprivation quintile | | | | |
| 1 | 0.96 | 0.91 | 1.01 | 0.108 |
| 2 | 0.96 | 0.91 | 1.01 | 0.141 |
| 3 | 1.00 | 0.94 | 1.06 | 0.961 |
| 4 | 0.99 | 0.93 | 1.04 | 0.000 |
| Missing | 1.01 | 0.95 | 1.07 | 0.809 |
| Age band | | | | |
| 0 - 28 days | 0.91 | 0.75 | 1.11 | 0.364 |
| 1 - 11 months | 1.18 | 1.14 | 1.21 | 0.000 |
| 0 – 4 years | - | - | - | - |
| 5 - 12 years | 0.80 | 0.78 | 0.85 | 0.000 |

*Figure 1: Forest plot of odds ratio of combined adverse events after initial oral antibiotics  prescriptions. the x value of 1 denotes no difference in odds ratio between the reference group and the group being compared. reference groups are -- age band: 1-4 years, ethnicity: white, sex: male, weight category: normal weight category.*

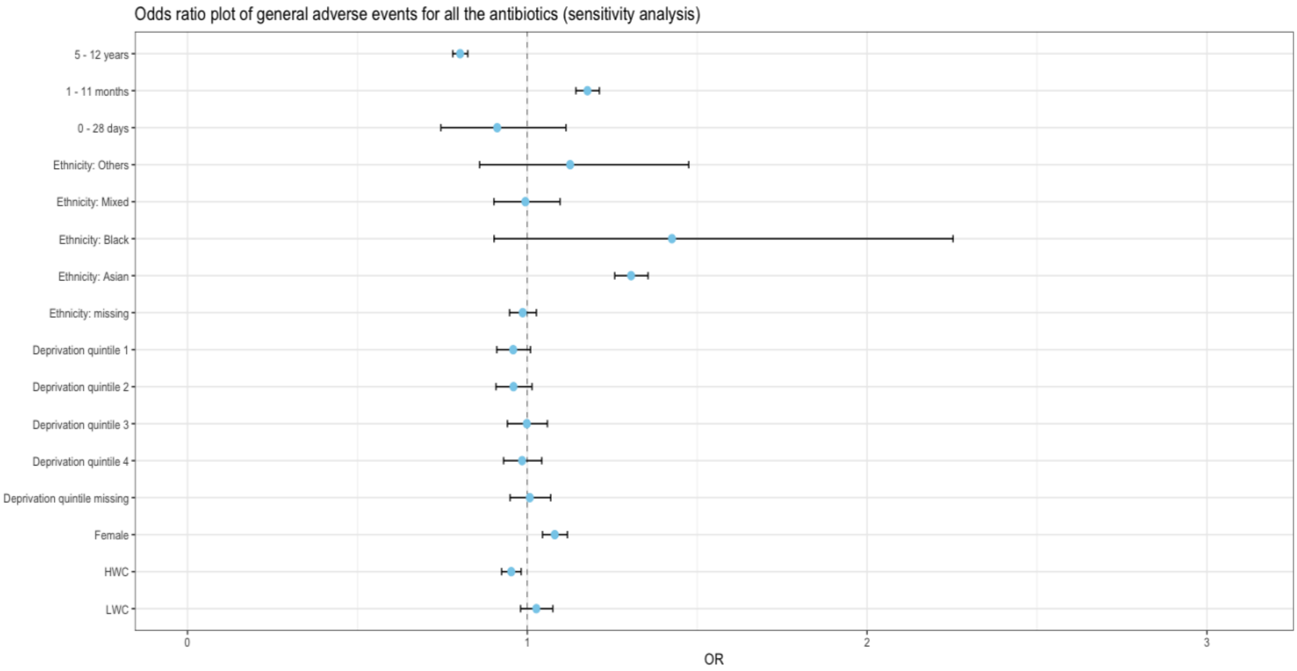

Supplement: online supplemental file 5 [file bmjpo-8-1-s005.pdf]
