# Supplementary material for: Association of child weight and adverse outcomes following antibiotic prescriptions in children: a national data study in Wales, UK
Source: BMJ Paediatr Open. 2024 Nov 28;8(1):e002831. doi: 10.1136/bmjpo-2024-002831 (PMC11605826; doi:10.1136/bmjpo-2024-002831)
Supplement: online supplemental file 6 [file bmjpo-8-1-s006.pdf]

Variables	General adverse events (combined)				Repeat antibiotics				Hospital/emergency admissions			
	OR	Lower CI	Upper CI	P values	OR	Lower CI	Upper CI	P values	OR	Lower CI	Upper CI	P values
Weight categories												
LWC	1.06	1.01	1.11	0.019	1.03	0.97	1.08	0.361	1.25	1.15	1.35	0.000
NWC	-	-	-	-	-	-	-	-	-	-	-	-
HWC	0.92	0.88	0.96	0.001	0.94	0.90	0.98	0.007	0.82	0.75	0.90	0.000
Sex												
Female	1.13	1.07	1.19	0.000	1.15	1.08	1.22	0.000	0.95	0.88	1.02	0.171
Male	-	-	-	-	-	-	-	-	-	-	-	-
Ethnic groups												
Asian	1.22	1.14	1.29	0.000	1.19	1.11	1.27	0.000	1.34	1.23	1.45	0.000
Black	1.37	0.68	2.74	0.381	1.38	0.63	3.05	0.423	1.06	0.70	1.59	0.797
Mixed	0.94	0.77	1.15	0.541	0.92	0.73	1.15	0.441	1.17	0.93	1.47	0.174
Missing	0.97	0.91	1.03	0.266	0.97	0.90	1.03	0.311	0.94	0.84	1.06	0.321
Other ethnicities	0.93	0.75	1.17	0.557	0.94	0.73	1.21	0.626	1.05	0.72	1.53	0.820
Whites	-	-	-	-	-	-	-	-	-	-	-	-
Deprivation quintiles												
1	1.00	0.93	1.09	0.941	0.97	0.89	1.07	0.589	1.14	1.01	1.29	0.038
2	0.99	0.90	1.08	0.775	0.96	0.87	1.07	0.455	1.13	0.99	1.29	0.072
3	0.96	0.89	1.03	0.230	0.95	0.87	1.02	0.178	0.99	0.86	1.14	0.909
4	0.99	0.91	1.07	0.714	0.99	0.91	1.08	0.821	0.91	0.78	1.05	0.188
5	-	-	-	-	-	-	-	-	-	-	-	-
Missing	0.99	0.91	1.07	0.746	0.93	0.85	1.03	0.183	1.20	1.04	1.38	0.013
Age bands												
0 - 28 days	0.60	0.45	0.81	0.001	0.41	0.28	0.60	0.000	1.73	1.13	2.65	0.011
1 - 11 months	0.97	0.91	1.04	0.422	0.87	0.81	0.95	0.001	1.52	1.38	1.66	0.000
0 - 4 years	-	-	-	-	-	-	-	-	-	-	-	-
5 - 12 years	0.76	0.73	0.81	0.000	0.81	0.77	0.85	0.000	0.54	0.50	0.59	0.000

Supplementary Table: Table showing the odds ratios of the risk factors for the respective adverse events (95% CI). LWC: Low Weight Category; NWC: Normal Weight Category; HWC: High Weight Category; Asian: Indian, Pakistani, Bangladeshi, Chinese, Any other Asian ethnic groups; Black: African, Caribbean, Any other black background; Mixed: White and Black Caribbean, White and Black African, White and Asian, Any other Mixed background; White: Any White Background (including Welsh, English, Scottish, Northern Irish, Irish, British), Gypsy, other White background; Other ethnicities: Arab and any other ethnic groups. Reference groups are -- age band: 1-4 years, ethnicity: white, sex: male, Weight categories: normal weigh category.
