# Supplementary material for: Association of child weight and adverse outcomes following antibiotic prescriptions in children: a national data study in Wales, UK
Source: BMJ Paediatr Open. 2024 Nov 28;8(1):e002831. doi: 10.1136/bmjpo-2024-002831 (PMC11605826; doi:10.1136/bmjpo-2024-002831)
Supplement: Supplementary file 7 [file bmjpo-2024-002831.reviewer_comments.pdf]

PEER REVIEW HISTORY

BMJ Paediatrics Open publishes all reviews undertaken for accepted manuscripts. Reviewers are asked to complete a checklist review form and are provided with free text boxes to elaborate on their assessment. These free text comments are reproduced below.

ARTICLE DETAILS

**TITLE (PROVISIONAL)**	Association of child weight and adverse outcomes following antibiotic prescriptions in children: A national data study in Wales, UK.
**AUTHORS**	Opatola, Ayodele; Seaborne, Mike; Kennedy, Jonathan; Hughes, Dyfrig; Laing, Hamish; Owen, Rhiannon; Tuthill, David; Bracchi, Robert; Brophy, Sinead

VERSION 1 - REVIEW

**REVIEWER NAME**	***Javier Ballesteros***
**REVIEWER AFFILIATION**	University of the Basque Country, Dept of Neuroscience - Psychiatry
**REVIEWER CONFLICT OF INTEREST**	
**DATE REVIEW RETURNED**	08-Jul-2024

**GENERAL COMMENTS**	Review of manuscript bmjpo-2024-002831<br><br>Based on record-linkage data, the manuscript analyzes the association of several variables (including weight but not specifically limited to it) with adverse outcomes related to antibiotic prescriptions in children aged 0 to 12 years. The analysis database includes 77,050 records after removing duplicate records and records that meet exclusion criteria. The following are my concerns regarding the current manuscript:<br><br>1. While the manuscript discusses the association of various variables (weight, sex, age, deprivation, and ethnicity) with the presence of adverse outcomes following antibiotic prescription and, thus, might be used as a predictive model in some sense, I am confused as to why the authors have shifted the focus of the manuscript to evaluating prescription recommendations for antibiotics in children based on age and weight. I do not believe the study design is suitable for addressing the authors' stated aims.<br>2. To further support the above point, the classification and regression training using the "caret" package in R is primarily utilized to generate reliable predictive models for elucidating specific outcomes (in this case, adverse events resulting from antibiotic consumption). It should be of interest to better understand the authors' results, and because of their reproducibility, to include a workflow template of the "caret" analyses as an appendix. The "caret" package offers a wide range of training models and methodological considerations, underscoring the significance of understanding the authors' selections for their analyses.<br>3. Since there are 52,480 not available data (NAs) for ethnicity and 4285 NAs for deprivation, Table 2 should also include the actual numbers for the analyses reported within each outcome category (general adverse events, repeat antibiotics, hospital/emergency admissions). Additionally, the values for the ORs and p-values

	reported are unnecessarily precise, two decimal places for ORs and three decimal places for p-values would be sufficient.<br>4. I do not understand why the authors included the analyses reported in Appendix 2 as a form of sensitivity analysis. The eligible population for the manuscript aims is not more than 300,000 possibly linked records but the 77,000 records which met the inclusion criteria.<br>5. As a final point, data in Table 2 is hard to interpret without a visual aid such as the one presented in Figure 1 for the excluded cohort (ORs with 95% confidence intervals).

VERSION 1 – AUTHOR RESPONSE

Reviewer 1:

1. Comment: “While the manuscript discusses the association of various variables (weight, sex, age, deprivation, and ethnicity) with the presence of adverse outcomes following antibiotic prescription and, thus, might be used as a predictive model in some sense, I am confused as to why the authors have shifted the focus of the manuscript to evaluating prescription recommendations for antibiotics in children based on age and weight. I do not believe the study design is suitable for addressing the authors' stated aims.”

• Response: The objective of the study has now been clarified to show that the analysis is not a predictive model. The following statement has been added to clarify this: “This study examines the association of adverse outcomes associated with oral antibiotic prescribing practices in pediatric primary care in Wales, with a specific emphasis on child weight” (page 4)

2. Comment: “To further support the above point, the classification and regression training using the "caret" package in R is primarily utilized to generate reliable predictive models for elucidating specific outcomes (in this case, adverse events resulting from antibiotic consumption). It should be of interest to better understand the authors’ results, and because of their reproducibility, to include a workflow template of the “caret” analyses as an appendix. The "caret" package offers a wide range of training models and methodological considerations, underscoring the significance of understanding the authors' selections for their analyses.”

• The study did not set out to develop a predictive model so we did not feel caret was appropriate for our work.

3. Comment: “Since there are 52,480, not available data (NAs) for ethnicity and 4285 NAs for deprivation, Table 2 should also include the actual numbers for the analyses reported within each outcome category (general adverse events, repeat antibiotics, hospital/emergency admissions). Additionally, the values for the ORs and p-values reported are unnecessarily precise, two decimal places for ORs and three decimal places for p-values would be sufficient.”

• We have now created a missing category for the variables with NA values with the number of missing values available in Table 1 of the supplementary materials. The OR values were also reported in 2 decimal places with the p values in 3 decimal places in Table 2.

4. Comment: “I do not understand why the authors included the analyses reported in Appendix 2 as a form of sensitivity analysis. The eligible population for the manuscript aims is not more than 300,000 possibly linked records but the 77,000 records which met the inclusion criteria.”

• A more detailed information on the reason for the sensitivity analysis was to assess the impact of collecting data above/below 30 days of index date; and inclusion of weight more than 112kg was

reported in the statistical analysis subsection of the methods section. This should address why the number of children in the analysis grew to more than 300,000.

5. Comment: “As a final point, data in Table 2 is hard to interpret without a visual aid such as the one presented in Figure 1 for the excluded cohort (ORs with 95% confidence intervals).”

• Odds ratio plots of the results have been provided for better understanding.

Summary of major changes:

1. The objective has been restructured for clarity

2. Figures containing Odds ratio plots have been added.

3. The total number of children in the analysis is slightly less with 71,541.

4. Deprivation quintile 1 did not have an increased tendency to cause adverse events as the lower limit of the odds ratio crossed the line of null effect. This was removed from the report.

5. Other changes such as the total number of adverse events and specific numbers were altered as well.

We hope that these revisions meet the expectations of the reviewers. Thank you again for the opportunity to improve our manuscript. We look forward to hearing from you regarding the next steps.

VERSION 2 – REVIEW

**REVIEWER NAME**	***Javier Ballesteros***
**REVIEWER AFFILIATION**	University of the Basque Country, Dept of Neuroscience - Psychiatry
**REVIEWER CONFLICT OF INTEREST**	
**DATE REVIEW RETURNED**	31-Oct-2024

**GENERAL COMMENTS**	The authors have improved the manuscript in the revision and have responded to the previous issues.
